# Prospective evaluation of low-dose multiphase hepatic computed tomography for detecting and characterizing hepatocellular carcinoma in patients with chronic liver disease

**DOI:** 10.1186/s12880-022-00947-7

**Published:** 2022-12-17

**Authors:** Eun Sun Choi, Jin Sil Kim, Jeong Kyong Lee, Hye Ah Lee, Seongyong Pak

**Affiliations:** 1 Department of Radiology and Medical Research Institute, College of Medicine, Ewha Womans University, Seoul, Korea; 2 Clinical Trial Center, Mokdong Hospital, Ewha Womans University, Seoul, Korea; 3 Department of Biomedical Engineering, Asan Medical Institute of Convergence Science and Technology, Asan Medical Center, University of Ulsan College of Medicine, Seoul, Republic of Korea

**Keywords:** Multidetector computed tomography, Radiation dose, Hepatocellular carcinoma, Low-dose CT, Ultralow-dose CT

## Abstract

**Background:**

Knowing the lowest acceptable radiation dose of multiphase hepatic CT may allow us to reduce the radiation dose for detecting HCC.

**Purpose:**

To prospectively assess the image quality and diagnostic performance of low-dose and ultra-low-dose multiphase hepatic computed tomography using a dual-source CT scanner.

**Methods:**

Three reconstructed different dose scan images (standard-dose, low-dose, and ultra-low-dose) of hepatic multiphase CT were obtained from 67 patients with a dual-source CT scanner. The image quality and the diagnostic performance of the three radiation dose CT scans of the hepatic focal lesion (≥ 0.5 cm) were analyzed by two independent readers using the Liver Imaging Reporting and Data System.

**Results:**

Qualitative image quality and signal-to-noise ratio were significantly different among the radiation doses (*p* < 0.001). In total, 154 lesions comprising 32 hepatocellular carcinomas (HCC) and 122 non-HCC were included. The sensitivities of SDCT, LDCT, and ULDCT were 90.6%(29/32), 81.3%(26/32), and 56.2%(18/32), respectively. The accuracies of SDCT, LDCT, and ULDCT were 98.1%(151/154), 96.1%(148/154), and 89.6%(138/154), respectively. On per-lesion analysis, SDCT and LDCT did not show significantly different sensitivity and accuracy in diagnosing HCC (*p* = 0.250 and 0.250).

**Conclusions:**

The diagnostic performance of dynamic hepatic LDCT with 33% reduced radiation dose in comparison to SDCT would be acceptable even though its image quality was qualitatively and quantitatively inferior. However, few HCCs could be overlooked. Therefore, with caution, radiation dose reduction by one-third could be implemented for follow-up CT scans for patients suspected of having HCC with caution and further studies are needed in the future.

## Introduction

Hepatic multiphase computed tomography (CT) is widely used to evaluate focal liver lesions in patients with chronic liver disease or liver cirrhosis [1, 2]. For such patients, a hepatic multiphase CT scan is usually used for evaluating hepatocellular carcinoma (HCC) or during follow-up after the treatment. In hepatic multiphase CT scans, two more phases, hepatic-arterial and delayed phases, are acquired in addition to pre-contrast and portal-venous phases, thus exposing the patient to twice as much radiation as routine abdominopelvic CT. Furthermore, patients with liver cirrhosis or chronic liver disease require several CT scans during their follow-up. Therefore, knowing the lowest acceptable radiation dose for detecting HCC in hepatic multiphase CT may reduce the radiation exposure rate in patients who must undergo hepatic multiphase CT studies.

In addition to common strategies to reduce CT radiation dose, such as iterative reconstruction, tube current modulation [3, 4], automatic exposure control [5, 6], and automated kilovolt modulation [7, 8], efforts were made to examine the possibility of reducing the radiation dose in abdominal CT scans [9–12]. However, these studies focused either on detecting focal liver lesions that were not HCC or on image analysis of single-phase abdominal CT scans. A recent study by Yoon et al. showed superior sensitivity and specificity of two phase low-dose hepatic CT compared to ultrasound (US) for detecting HCC in a high-risk group [13], emphasizing the role of multiphase hepatic CT in the surveillance setting.

In our prospective study, we aimed to assess the image quality and diagnostic performance of hepatic multiphase CT scans of different radiation doses for detecting HCC in patients with chronic liver disease.

## Materials and methods

### Patient population

**Fig. 1:**
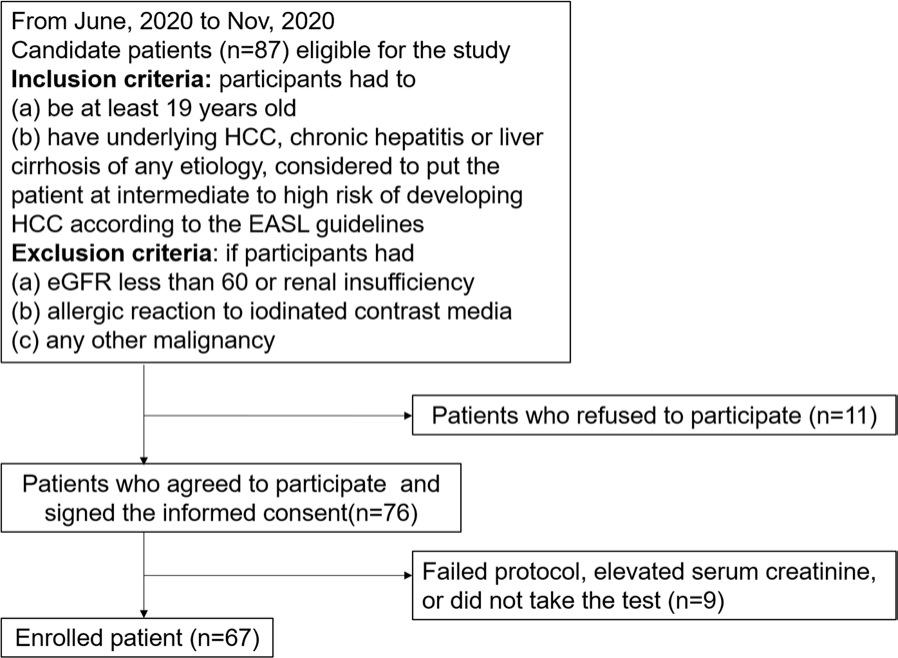
Patient inclusion and exclusion criteria. (EASL, European Association for the Study of the Liver)

This prospective study was approved by the institutional review board of our institution, and written informed consent was obtained from all patients. Our inclusion criteria were as follows: participants should (a) be at least 18 years old; (b) have underlying HCC, chronic hepatitis, or cirrhosis of any etiology that is considered to put the patient at intermediate to high risk of developing HCC according to the European Association for the Study of the Liver guidelines [14]; and (c) have signed informed consent. Exclusion criteria were as follows: participants had (a) estimated glomerular filtration rate < 60 or renal insufficiency; (b) allergic reaction to iodinated contrast media; and (c) any other malignancy. Between June 2020 and November 2020, a total of 87 eligible patients were asked to participate in the study. Out of the 76 patients who agreed to participate, 9 patients were not included due to failure in following protocol, canceled CT examination, or elevated creatinine levels at the time of CT examination (Fig. 1). Finally, 67 patients were included in this study.

### CT scanning protocol and image reconstruction

All patients in this study were examined once with a 256-detector row CT scanner (Somatom definition flash; Siemens Healthcare, Forchheim, Germany). We used the dual-source mode; “tube A” was responsible for two-thirds (66.7%) of the total radiation dose and “tube B” for one-third (33.3%) of the total radiation dose. Contrast-enhanced CT was performed with automated tube current modulation (CareDose4D, Siemens Healthcare), and imaging parameters were as follows: 128 × 0.67-mm collimation, 0.8–1.0 pitch, 3-mm reconstruction interval, 100 kVp, and 90–140 mAs. In the phantom study, dual-source mode showed radiation dose approximately 7–8% higher than the single-tube CT. Therefore, we lowered the total dose by 8% from the default setting. Unenhanced scans were initially obtained. Thereafter, 120 mL of contrast medium (Iohexol 350 mg I/mL; 600 mg I/kg based on 70 kg) was injected intravenously at 3 mL/s using an automatic power injector. The arterial phase was obtained using the bolus triggering technique 12 s after a trigger threshold of 100 HU at the abdominal aorta. Portal venous and delayed phases were obtained 80–90 s and 180 s after contrast medium administration, respectively. The dose-length product was automatically calculated, and the effective dose was estimated using a coefficient of 0.015 [15].

Images were reconstructed into following three dose sets: SDCT (from both tube A and B), LDCT (from tube A, 66.7%), and ULDCT (from tube B, 33.3%). A partial model-based iterative reconstruction technique from ADMIRE 3, known to deliver the highest reconstructed image quality of abdominal CT, was selected for the reconstruction method [9, 16, 17].

### Qualitative and quantitative image quality assessment

The overall image qualities of arterial phase and portal phase of SDCT, LDCT, and ULDCT scans were graded on a 5-point Likert scale defined as follows: 1, issues affecting diagnostic information; 2, major issues affecting visualization of major structures but diagnosis still possible; 3, minor issues possibly interfering with diagnostic decision making; 4, minor issues not interfering with diagnostic decision making; and 5, excellent image quality without related issues of concern. Scores 1 and 2 were considered unacceptable for diagnostic purposes.

Noise values [standard deviations (SDs) in Hounsfield units (HU)] were measured by drawing a circular region of interest (ROI) measuring about 1–3 cm^2^ on transverse images of SDCT, LDCT, and ULDCT scans. The noise was determined as the mean SD of the three measurements of the anterior and posterior abdominal wall fat at the left renal vein [18] by a single-blinded reader (E.S.C, with 2 years of experience in abdominal radiology). Liver ROI was measured at the homogeneous area between middle and right hepatic veins at the portal vein level; SNR and CNR were calculated for each image dataset using the following equations: SNR = (mean HU of liver/noise) [19] and CNR = (mean HU of focal arterial enhancing lesion − mean HU of the liver (arterial))/(SD_liver) [20]. The mean HU of liver and noise for SNR were measured in the pre-contrast phase of each dose scan. Values for CNR of HCC (n = 32) were measured in the arterial phase.

### Focal lesion detection and characterization

Two independent board-certified radiologists (J.K.L, with more than 20 years of experience in body imaging and J.S.K, with 10 years of experience in body imaging) performed focal lesion detection of three primary series in three reading sessions. The evaluation of overall image quality and focal lesion characterization were performed together. For the focal liver lesion, we evaluated the detection rate, accuracy, sensitivity, and specificity. If the patient had multiple lesions, we set the limit of up to 10 focal lesions per patient. Between each reading session, we had a 4-week washout period to avoid recall bias. Focal lesions ≥ 0.5 cm were included. Wedge-shaped arterial enhancing lesions were considered arterioportal (A-P) shunts, and A-P shunts were not included for focal lesion evaluation. The LI-RADS (v2018) score was assigned for the focal liver lesion [2]. LI-RADS scores of 4 and 5 were considered to be indicative of HCC. The maximum diameter of the focal liver lesion was measured on axial images. Undetected lesions were considered negative.

### Reference standards

The principal investigator (J.S.K, with 10 years of experience in abdominal radiology) subsequently established the reference standard using all available clinical data, pathology results (surgery or biopsy), and cross-sectional imaging examinations. HCC was indicated by (1) LR-4 and LR-5 lesions on SD four-phase hepatic CT or follow-up gadoxetic acid-enhanced liver MRI or (2) tumor staining on cone-beam CT for TACE followed by compact lipiodol uptake. Imaging criteria for benign lesions were based on imaging features plus stability in a separate CT or MR examination performed at least 6 months before or after the index CT examination.

### Statistical analysis

All statistical analyses were performed using commercially available statistical software (IBM SPSS Statistics for Windows, v. 26.0; IBM, Armonk, NY; or MedCalc, v. 19.2.1; MedCalc, Marikerke, Belgium). Two-tailed *p* values < 0.05 were considered statistically significant.

Lesion detection and diagnostic performance were analyzed on a per lesion basis. Per-lesion sensitivity, specificity, and accuracy values were calculated based on the reference standard, and the results of liver lesion detection were compared between the sets using the McNemar test. Because the modified Barcelona Clinical Liver Cancer determined lesions < 2 cm as very early stage, we analyzed the lesions by categorizing them into lesions < 2 cm and ≥ 2 cm [14]. If any reader detected a lesion for SDCT, LDCT, or ULDCT, then that lesion was considered detected for that dose. If any reader classified a lesion as HCC, that lesion was considered HCC for SDCT, LDCT, or ULDCT. This approach assesses the visibility and diagnostic performance of a lesion among scans along while limiting the differences between the readers [10].

Dose parameters, quantitative measurements including CNR and SNR, and qualitative analysis assessing the image quality of the hepatic arterial phase and portal venous phase were compared by ANOVA with *post-hoc* Bonferroni corrections. For qualitative analysis of arterial phase image and portal phase image, interobserver variability was evaluated using an average-measure 2-way random-effects intraclass correlation coefficient (ICC) for qualitative analysis regarding image quality [21]. Agreement was considered to be poor (< 0.40), fair to good (0.40–0.75) or excellent (> 0.75) [22].

## Results

The demographics of the 67 participants and the mean effective dose of each radiation dose are organized in Table 1.

**Table 1:** Demographic of the study population (n = 67)

Parameters	Values
Sex (men:women), n	47:20
Age, years	
Men	62.2 ± 11.6 (40–86)^†^
Women	62.2 ± 13.3 (30–83)^†^
Underlying disease, % (n/N)	
Chronic hepatitis B	40.3 (27/67)
Chronic hepatitis C	10.4 (7/67)
Alcoholic liver disease	19.4 (13/67)
Cryptogenic	29.9 (20/67)
Laboratory findings	
Albumin, g/dL	4.3 ± 0.5 (2.6–5.2)^†^
Total bilirubin, mg/dL	0.9 ± 0.7 (0.3–4.1)^†^
INR	1.1 ± 0.2 (0.9–1.9)^†^
Platelet count, × 10^3^/mm^3^	146.1 ± 63.8 (42–317)^†^
AFP, ng/mL	329.4 ± 1699.6 (1.6–12,327)
Body weight, kg	65.1 ± 12.1 (39.6–96.6)^†^
Mean body mass index, kg/m^2^	24.1 ± 3.8 (15.3–35.3)^†^
DLP, mGycm	
Standard dose	848.8 ± 208.6 (423.0–1432.0)^†^
Low dose	568.7 ± 139.7 (296.1–1002.4)^†^
Ultra-low dose	280.1 ± 68.8 (126.9–429.6)^†^
Effective dose (mSv)	
Standard dose	12.7 ± 3.1 (6.3–21.5)^†^
Low dose	8.5 ± 2.1 (4.4–15.0)^†^
Ultra-low dose	4.2 ± 1.0 (1.9–6.4)^†^

*INR* international normalized ratio, *AFP* alpha-fetoprotein, *DLP* dose length product

^†^Data are represented as mean and standard deviation. Minimum and maximum values in parenthesis with hyphen. ‘n’ indicates the number of people in that parameter, and ‘N’ indicates the total number of enrolled patients. Unit of each parameter is noted after the comma of each criterion

In the qualitative assessment of both overall image quality, higher dose scans scored significantly better than lower dose scans (*p* < 0.001, Table 2). For the overall image quality scores of the arterial phase and portal phase, the agreement between the two observers showed fair to good agreement (ICC = 0.679 and 0.537, respectively). SNR was significantly different among the different radiation dose scans (*p* < 0.001, Table 3). The CNR of LDCT was not significantly different from that of SDCT (*p* = 0.112) or that of ULDCT (*p* = 0.075) (Table 3).

**Table 2:** Qualitative image quality analysis according to dose level

	SDCT	LDCT	ULDCT	*p* Value for pairwise comparison
Overall image quality (Arterial phase)				
R1	4.4 ± 0.5 (4–5) (median: 4, IQR, 4–5)	3.8 ± 0.5 (3–5) (median: 4, IQR, 4–4)	2.7 ± 0.6 (2–4) (median: 3, IQR, 2–3)	< 0.001^+^ SD versus LD, < 0.001 SD versus ULD, < 0.001 LD versus ULD, < 0.001
R2	4.3 ± 0.7 (2–5) (median: 4, IQR, 4–5)	3.9 ± 0.6 (3–5) (median: 4, IQR, 4–4)	3.1 ± 0.4 (2–4) (median: 3, IQR, 3–3)	
Average	4.4 ± 0.6 (2–5) (median: 4, IQR, 4–5)	3.9 ± 0.5 (3–5) (median: 4, IQR, 4–4)	2.9 ± 0.6 (2–4) (median: 3, IQR, 3–3)	
Overall image quality (Portal phase)				
R1	4.5 ± 0.5 (4–5) (median: 4, IQR, 4–5)	3.9 ± 0.5 (3–5) (median: 4, IQR, 4–4)	2.8 ± 0.6 (2–4) (median: 3, IQR, 2–3)	< 0.001^+^ SD versus LD, < 0.001 SD versus ULD, < 0.001 LD versus ULD, < 0.001
R2	4.9 ± 0.5 (2–5) (median: 5, IQR, 5–5)	4.6 ± 0.6 (4–5) (median: 5, IQR, 4–5)	3.5 ± 0.6 (2–5) (median: 3, IQR, 3–4)	
Average	4.3 ± 0.7 (2–5) (median: 4, IQR, 4–5)	4.2 ± 0.6 (3–5) (median: 4, IQR, 4–5)	3.1 ± 0.7 (2–5) (median: 3, IQR, 2–3)	

Data are mean and standard deviation with minimum and maximum values in parentheses. ^+^indicates the *p* value in ANOVA analysis

*SDCT* standard-dose CT, *LDCT* low-dose CT, *ULDCT* ultra-low-dose CT, *R1* Reader 1, *R2* Reader2, *IQR* interquartile range

**Table 3:** Quantitative assessment according to dose level on the pre-contrast image

	SDCT	LDCT	ULDCT	*p* Value for pairwise comparison
SNR	6.2 ± 1.5	5.1 ± 1.1	4.4 ± 1.1	< 0.001^+^ SD versus LD, < 0.001 SD versus ULD, < 0.001 LD versus ULD, 0.002
CNR	3.8 ± 2.2	3.1 ± 2.0	2.3 ± 1.7	0.012^+^ SD versus LD, 0.112 SD versus ULD, 0.001 LD versus ULD, 0.075

*SNR* signal-to-noise ratio, *CNR* contrast-to-noise ratio

The data are the mean ± SD. ^+^indicates the *p* value in ANOVA analysis

Out of a total of 67 enrolled patients, 154 lesions (32 HCC and 122 non-HCC) were included from 57 patients. The following patients were not included in the diagnostic performance analysis: patients (1) without focal lesion (n = 4), (2) without follow-up image more than 6 months (n = 5), or (3) with HCC involving more than hemi-liver for focal lesion evaluation (n = 1). The mean size of HCC was 1.8 cm ± 1.7 cm (0.6–6.6 cm). The mean size of benign lesions was 1.0 cm ± 0.6 cm (0.5–4.7 cm). The mean size of the total lesion was 1.1 cm ± 0.9 cm (0.5–6.6 cm). In SDCT scans, all HCCs were detected. In LDCT, 93.7% HCCs were detected, and in ULDCT, only 78.1% were detected (Table 4). The detectability of LDCT was not significantly different from that of SDCT (*p* = 0.500) or ULDCT (*p* = 0.063). However, the detectability of ULDCT was significantly different from that of SDCT (*p* = 0.016). In the subgroup analysis of the 28 positive lesions (HCCs) measuring < 2 cm, LDCT detected two lesions fewer than SDCT, and the difference was not statistically significant (*p* = 0.500); however, ULDCT detected seven lesions fewer than SDCT, and the difference was statistically significant (*p* = 0.016). This aspect did not significantly differ between ULDCT and LDCT (*p* = 0.063). All lesions ≥ 2 cm were detected in SDCT, LDCT, and ULDCT.

**Table 4:** The detection rate of focal lesion according to size (HCC, n = 32)

	SDCT	LDCT	ULDCT
< 20 mm			
R1	96.4% (27/28)	82.1% (23/28)	64.3% (18/28)
R2	92.9% (26/28)	85.7% (24/28)	71.4% (20/28)
Total	100% (28/28)	92.9% (26/28)	75.0% (21/28)
≥ 20 mm			
R1	100% (4/4)	100% (4/4)	100% (4/4)
R2	100% (4/4)	100% (4/4)	100% (4/4)
Total	100% (4/4)	100% (4/4)	100% (4/4)
Total	100% (32/32)	93.7% (30/32)	78.1% (25/32)

The parenthesis indicates the number of HCC patients detected by the reader in that size criteria over the total number of HCC patients in the size criteria. Total included lesion detected by either reader

*SDCT* standard-dose CT, *LDCT* low-dose CT, *ULDCT* ultra-low-dose CT, *R1* Reader 1, *R2* Reader2

**Fig. 2:**
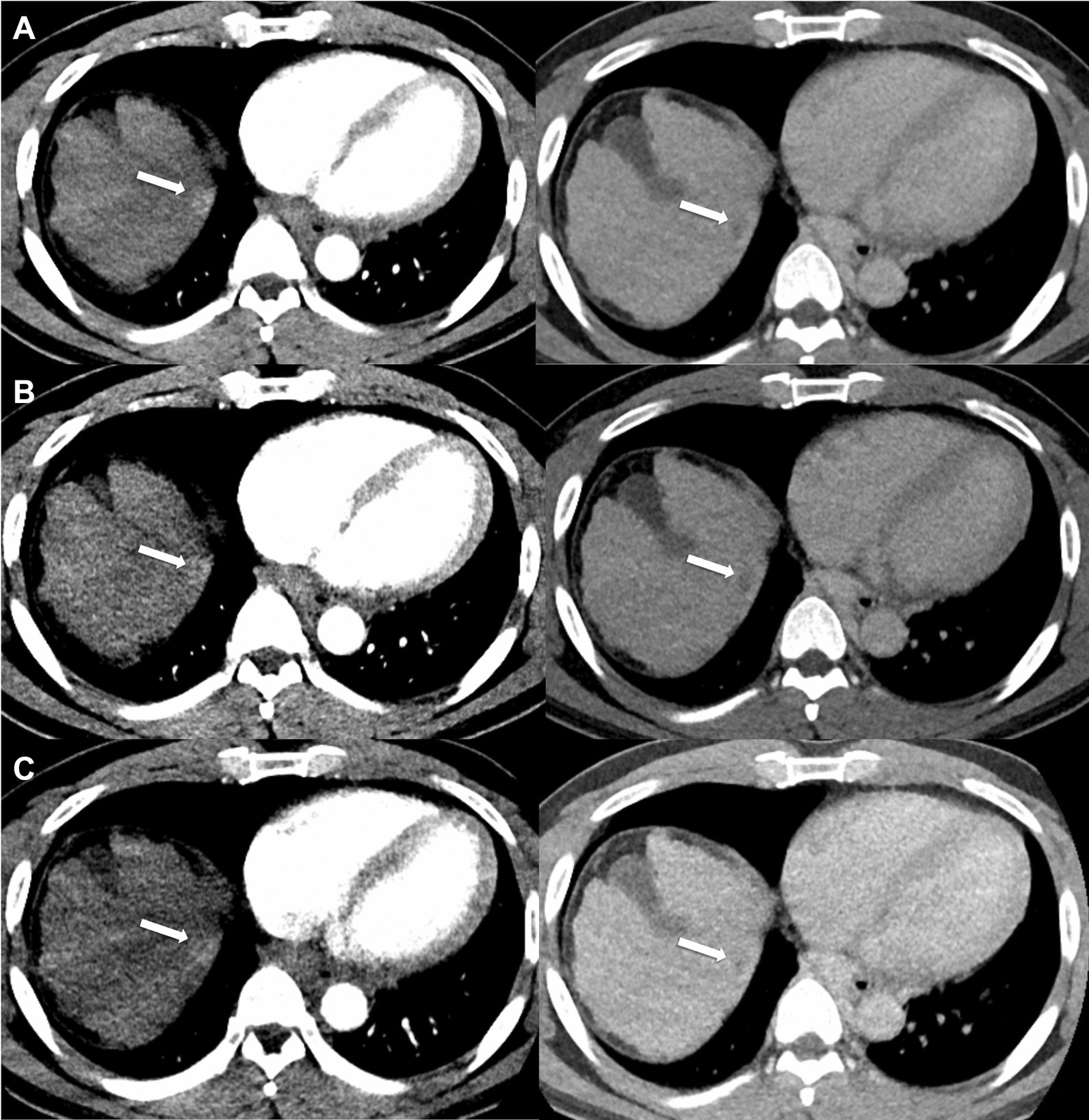
A 1.5-cm sized hepatocellular carcinoma in a 45-year-old man with 28.2 kg/m^2^ body mass index. **A** Standard dose CT (17.1 mSv) arterial and delayed phase, **B** low-dose CT (12.0 mSv) arterial and delayed phase, and **C** ultra-low-dose CT (5.1 mSv) arterial and delayed phase. On SDCT (**A**), both readers detected the lesion as hepatocellular carcinoma. However, reviewer 1 could not detect the lesion on LDCT (**B**) and ULDCT (**C**). Reviewer 2 detected the lesion but was characterized as benign (LI-RADS 3)on LDCT (**B**) and ULDCT (**C**). Reviewer 1 and reviewer 2 rated the overall image quality as 5 and 4 respectively for SDCT (**A**), 3 and 4 respectively for LDCT (**B**), and 2 and 4 respectively for ULDCT(**C**)

**Fig. 3:**
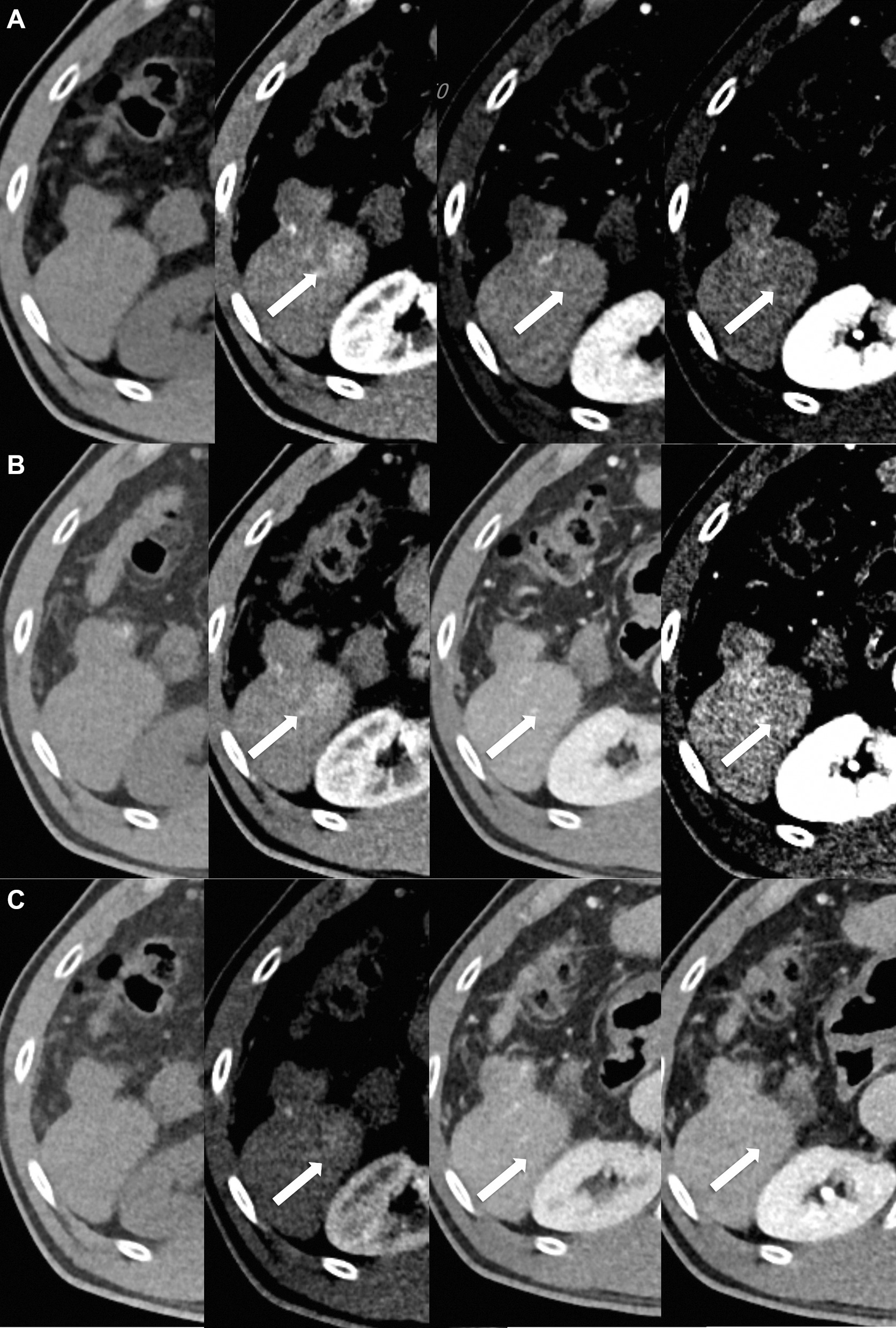
A 1.7-cm sized hepatocellular carcinoma in a 60-year-old man with 22.9 kg/m^2^ body mass index. **A** In standard-dose CT (9.3 mSv), reviewer 1 gave a LI-RADS score of 5, and reviewer 2 gave a score of 3. **B** In low-dose CT (6.5 mSv), both reviewers gave a LI-RADS score of 3. **C** In ultra-low-dose CT (2.8 mSv), both reviewers did not detect the lesion

**Fig. 4:**
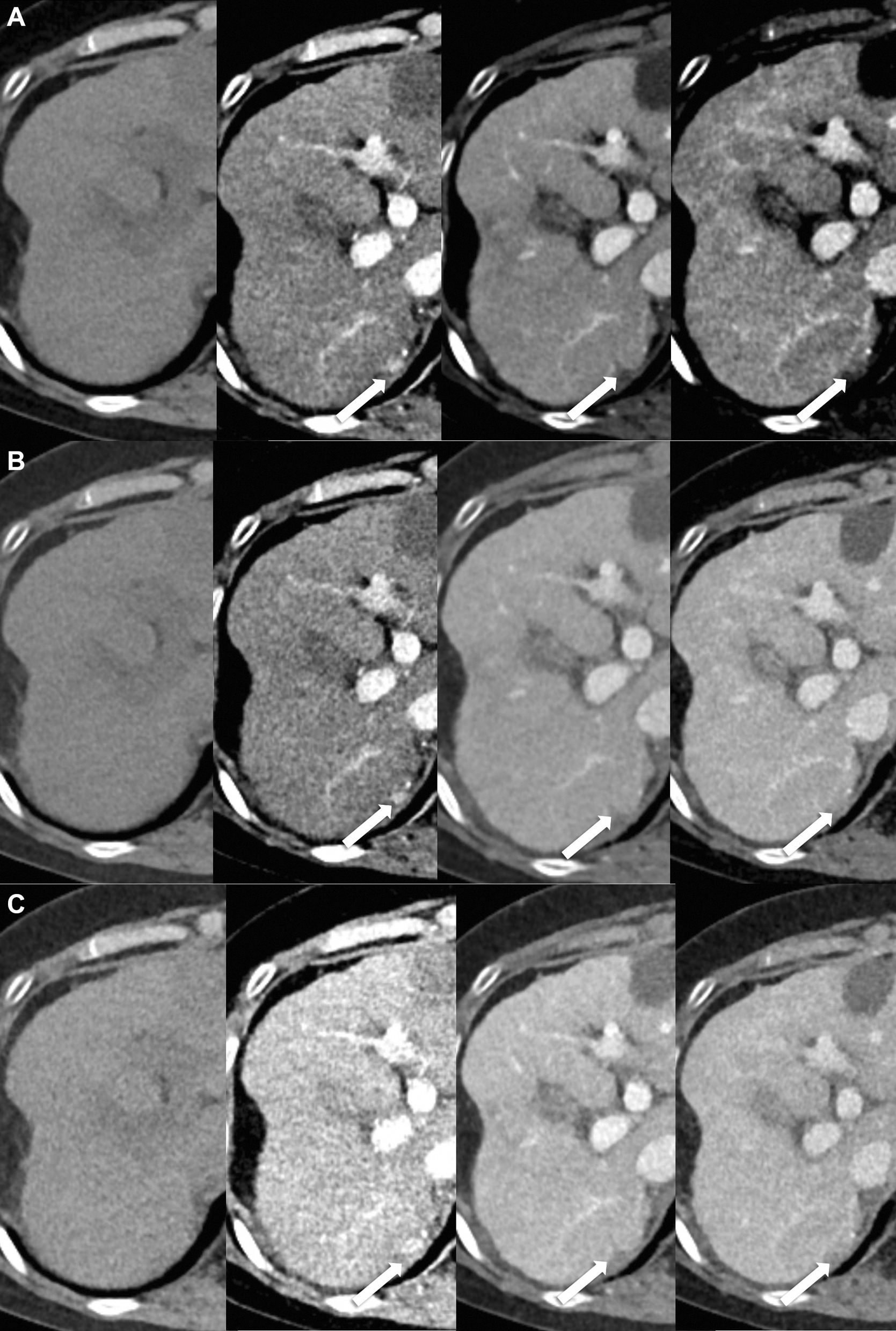
A 0.9-cm sized hepatocellular carcinoma in a 65-year-old woman with 20 kg/m^2^ body mass index. **A** In standard-dose CT (8.3 mSv), both reviewers gave a LI-RADS score of 4. **B** In low-dose CT (5.8 mSv), reviewer 1 gave a LI-RADS score of 4 but reviewer 2 could not detect the lesion. **C** Both reviewers could not detect the lesion in ultra-low-dose CT (2.5 mSv)

On per-lesion analysis, SDCT and LDCT did not show significantly different sensitivity and accuracy in diagnosing HCC (*p* = 0.250 and 0.250, respectively). In contrast, ULDCT scans showed significant difference in sensitivity and accuracy when compared to SDCT (*p* = 0.001 and *p* < 0.001, respectively) and LDCT (*p* = 0.022 and *p* = 0.006, respectively) (Tables 5, Figs. 2, 3, 4). In the subgroup analysis of lesions measuring < 2 cm, there was no statistical difference in sensitivity (*p* = 0.250) and accuracy (*p* = 0.250) between SDCT and LDCT. Further, the sensitivity of ULDCT was significantly different from both SDCT (*p* = 0.002) and LDCT (*p* = 0.039), and concurrently, the accuracy of ULDCT was significantly different from that of SDCT (*p* < 0.001) and LDCT (*p* = 0.012). In the subgroup analysis of lesions > 2 cm, there was no significant difference in diagnostic performance among the different dose scans.

**Table 5:** Characterization of focal lesion (32 HCC and 122 non-HCC) according to size

	SDCT				
	Sensitivity	Specificity	PPV	NPV	Accuracy
< 20 mm					
R1	85.2 (23/27)	100 (117/117)	100 (23/23)	96.7 (117/121)	97.2 (140/144)
R2	85.2 (23/27)	100 (117/117)	100 (23/23)	96.7 (117/121)	97.2 (140/144)
At least one	88.9 (24/27)	100 (117/117)	100 (24/24)	97.5 (117/120)	97.9 (141/144)
> 20 mm					
R1	100 (5/5)	100 (5/5)	100 (5/5)	100 (5/5)	100 (10/10)
R2	100 (5/5)	100 (5/5)	100 (5/5)	100 (5/5)	100 (10/10)
At least one	100 (5/5)	100 (5/5)	100 (5/5)	100 (5/5)	100 (10/10)
Total					
R1	87.5 (28/32)	100 (122/122)	100 (28/28)	96.8 (122/126)	97.4 (150/154)
R2	87.5 (28/32)	100 (122/122)	100 (28/28)	96.8 (122/126)	97.4 (150/154)
At least one (95% C.I)	90.6 (29/32) (75.0–98.0)	100 (122/122) (97.0–100)	100 (29/29)	97.6 (122/125) (93.3–99.2)	98.1 (151/154) (94.4–99.6)

	LDCT				
	Sensitivity	Specificity	PPV	NPV	Accuracy
< 20 mm					
R1	74.1 (20/27)	100 (117/117)	100 (20/20)	94.4 (117/124)	95.1 (137/144)
R2	74.1 (20/27)	100 (117/117)	100 (20/20)	94.4 (117/124)	95.1 (137/144)
At least one	77.8 (21/27)	100 (117/117)	100 (21/21)	95.1 (117/123)	95.8 (138/144)
> 20 mm					
R1	100 (5/5)	100 (5/5)	100 (5/5)	100 (5/5)	100 (10/10)
R2	100 (5/5)	100 (5/5)	100 (5/5)	100 (5/5)	100 (10/10)
At least one	100 (5/5)	100 (5/5)	100 (5/5)	100 (5/5)	100 (10/10)
Total					
R1	78.1 (25/32)	100 (122/122)	100 (25/25)	94.6 (122/129)	95.5 (147/154)
R2	78.1 (25/32)	100 (122/122)	100 (25/25)	94.6 (122/129)	95.5 (147/154)
At least one (95% C.I)	81.3 (26/32) (63.6–92.8)	100 (122/122) (97.0–100)	100 (26/26)	95.3 (122/128) (90.8–97.7)	96.1 (148/154) (91.7–98.6)

	ULDCT				
	Sensitivity	Specificity	PPV	NPV	Accuracy
< 20 mm					
R1	48.1 (13/27)	98.3 (115/117)	86.7 (13/15)	89.1 (115/129)	88.9 (128/144)
R2	40.7 (11/27)	100 (117/117)	100 (11/11)	88.0 (117/133)	88.9 (128/144)
At least one	51.9 (14/27)	98.3 (115/117)	87.5 (14/16)	89.8 (115/128)	89.6 (129/144)
> 20 mm					
R1	80 (4/5)	100 (5/5)	100 (4/4)	100 (5/6)	90.0 (9/10)
R2	80 (4/5)	100 (5/5)	100 (4/4)	100 (5/6)	90.0 (9/10)
At least one	80 (4/5)	100 (5/5)	100 (4/4)	100 (5/6)	90.0 (9/10)
Total					
R1	53.1 (17/32)	98.4 (120/122)	89.5 (17/19)	89.7 (120/135)	89.0 (137/154)
R2	46.9 (15/32)	100 (122/122)	100 (15/15)	88.4 (122/139)	89.0 (137/154)
At least one (95% C.I)	56.2 (18/32) (37.7–73.6)	98.4 (120/122) (94.2–99.8)	90.0 (18/20) (68.8–97.3)	89.6 (120/134) (85.3–92.7)	89.6 (138/154) (83.7–93.9)

Values are in percentage and the fraction noted in the parenthesis indicates the number of positive lesions over the total lesions. At least one indicates the positive lesions detected or diagnosed either by reviewer 1 or 2

*SDCT* standard-dose CT, *LDCT* low-dose CT, *ULDCT* ultra-low-dose CT, *R1* Reader 1, *R2* Reader2, *PPV* positive predictive value, *NPV* negative predictive value, *CI* confidence interval

## Discussion

The diagnostic performance of the LDCT (i.e., 66.7% radiation dose compared to SDCT) was not significantly affected (accuracy, SDCT vs. LDCT, 98.1% vs. 96.1%, *p* = 0.250). However, we found SDCT to have superior objective and subjective image quality (SNR, SDCT vs. LDCT, 6.2 ± 1.5 vs. 5.1 ± 1.1, *p* < 0.001; overall image quality, SDCT vs. LDCT, 4.4 ± 0.6 vs. 3.9 ± 0.5, *p* < 0.001). However, ULDCT showed significantly lower accuracy (89.6%) in comparison to SDCT (*p* < 0.001).

Patients with chronic liver disease or liver cirrhosis are required to undergo multiple hepatic multiphase CT scans over their lifetime for the evaluation of HCC, post-treatment response, or new lesion detection. Compared to routine abdominal CT, hepatic multiphase CT requires four phases, thereby increasing the radiation dose [23, 24]. We believe this study will help recognize the possibility of reducing the radiation dose.

Despite significant reductions in subjective and objective image qualities, our study showed that LDCT offered similar diagnostic performance as SDCT for a focal liver lesion in patients with chronic liver disease or liver cirrhosis. These results are consistent with those of previous studies showing similar accuracy of LDCT (effective dose, 2.6 mSv) for focal abdominal lesions including hepatic lesions in the portal venous phase [16]. Our findings are also in line with previous reports suggesting that a moderately aggressive dose reduction LDCT (mean effective dose, 2 mSv) could be more appropriate for oncological follow-up while ensuring that focal liver lesions are not overlooked [25]. Although LDCT (mean dose length product, 324.9 mGy cm) exhibited low sensitivity for the evaluation of small, low-contrast liver lesions (colorectal cancer hepatic metastasis) using iterative reconstruction in a previous study [10], LDCT for the evaluation of HCC using liver dynamic protocol showed a lower effective dose with increased sensitivity. This is because the average size of the lesion in the previous study was approximately 0.7 cm, and lesions as small as 0.2 cm were included. Moreover, compared to colorectal metastasis, hepatocellular carcinoma is usually encapsulated and shows better contrast in CT scans [26–28]. Therefore, despite the lower subjective and objective image quality, LDCT showed reasonable diagnostic performance with no significant discrepancy compared with SDCT.

Many studies proved iterative reconstruction to be useful in reducing the radiation dose [29, 30]. In a study conducted by Nakaura et al., an 80-kVp protocol enabled the reduction of the effective dose by 51% during the hepatic arterial phase and by 48% during the portal venous phase. Although our study did not directly compare the filtered back projection and iterative reconstruction, using the iterative reconstruction technique for reconstruction, we saw a similar accuracy between LDCT and  SDCT (96.1% vs. 98.1%). Considering the radiation dose of the arterial phase to be 5.6 mSv based on the previous study by Nakaura et al., the most recent CT machine requires approximately 2.3 mSv (at our institution), which is one-fourth of 8.9 mSv.

Yoon et al. [13] studied the feasibility of a two-phase LDCT in HCC surveillance and highlighted the superiority of low-dose two-phase CT scans to US in detecting HCC in high-risk patients. In comparison to Yoon et al.’s study, our study showed a similar radiation dose (7.9 ± 3.0 mSv) and included patient population with a similar body mass index. Although our study was not designed in the surveillance setting and therefore included patients with known HCC, the diagnostic performance of LDCT was still acceptable in comparison to SDCT, and the sensitivity of LDCT was similar to that reported in Yoon et al.’s study (83.3% vs. 81.3%). However, there was reduced sensitivity in the LDCT by 7.1% in lesions less than 2 cm (Table 4) despite the small number. Also, wide confidence intervals was demonstrated in Table 5. Since the theoretical risk from increased radiation doses does not warrant missing HCC, for firm conclusions, a large-scale study is needed to support our findings in the future.

In a study by Khawaja et al. [31], ULDCT showed a significant reduction in diagnostic performance. Such findings contradict the past study which showed no significant difference in the detection rate for ULDCT (< 0.9 mSv) and SDCT. They included only 14 liver lesions and also included lesions in other abdominal organs. Because they did not specify the size of the lesion, it may be possible that they only included relatively large lesions. Such drastic reduction in radiation dose may alter the HCC detection rate and specificity, leading to further unnecessary workup. Therefore, ULDCT could not be appropriate for the evaluation of HCC in patients with chronic liver disease or liver cirrhosis.

Our study has several limitations. First, blinding of readers to different dose scans could be limited by image qualities being significantly different among SDCT, LDCT, and ULDCT. Regardless, the diagnostic performance was less affected because the reference standard was blinded. In addition, four-week washout period may not be sufficient to avoid recall bias. Second, we assumed that the reconstructed SD image of the dual-source mode be equivalent to that of the single-source standard image. However, there might exist some differences between the image quality of the reconstructed SD image using the dual-source mode and the image obtained from a single tube with true dose because in the phantom study where we set effective mAs in the two tubes, the dual-source mode showed a 7% increase in the total radiation dose compared to our single-tube CT, and thus, we reduced the default dose by such a ratio. Third, the specificity in the SD scan was 100% because we excluded lesions that were either not followed-up on or were not confirmed. Additionally, because the benign lesion was evaluated based on SDCT, the specificity was relatively higher in ULDCT where the lesion was not at all detected. Fourth, we included a small number of HCCs because patients were enrolled without confirming the presence of HCC as a prospective study. A large-scale study is needed to support our findings in the future.

In conclusion, the diagnostic performance of dynamic hepatic LDCT with 33% reduced radiation dose in comparison to SDCT would be acceptable even though its image quality was qualitatively and quantitatively inferior. However, few HCCs could be overlooked. Therefore, with caution, radiation dose reduction by one-third could be implemented for follow-up CT scans for patients suspected as having HCC and further studies are needed in the future.

## Data Availability

The datasets analyzed in the current study are available from the corresponding authors on reasonable request.

## Abbreviations

CNR: Contrast-to-noise ratio
CT: Computed tomography
HCC: Hepatocellular carcinoma
EASL: European Association for the Study of the Liver hepatocellular carcinoma
LD: Low dose
LDCT: Low dose computed tomography
LI-RADS: Liver Imaging Reporting and Data System
SD: Standard dose
SDCT: Standard dose computed tomography
SNR: Signal-to-noise ratio
TACE: Trans-arterial chemoembolization
ULD: Ultra low dose
ULDCT: Ultra low dose computed tomography
